# Accuracy–Power Controllable LiDAR Sensor System with 3D Object Recognition for Autonomous Vehicle

**DOI:** 10.3390/s20195706

**Published:** 2020-10-07

**Authors:** Sanghoon Lee, Dongkyu Lee, Pyung Choi, Daejin Park

**Affiliations:** 1 Carnavicom Co., Ltd., Incheon 21984, Korea; shlee@carnavi.com; 2 School of Electronic and Electrical Engineering, Kyungpook National University, Daegu 41566, Korea; dklee1215@knu.ac.kr (D.L.); p0choi@ee.knu.ac.kr (P.C.)

**Keywords:** LiDAR sensor processor, low-power circuit design, 3D object recognition, autonomous vehicle

## Abstract

Light detection and ranging (LiDAR) sensors help autonomous vehicles detect the surrounding environment and the exact distance to an object’s position. Conventional LiDAR sensors require a certain amount of power consumption because they detect objects by transmitting lasers at a regular interval according to a horizontal angular resolution (HAR). However, because the LiDAR sensors, which continuously consume power inefficiently, have a fatal effect on autonomous and electric vehicles using battery power, power consumption efficiency needs to be improved. In this paper, we propose algorithms to improve the inefficient power consumption of conventional LiDAR sensors, and efficiently reduce power consumption in two ways: (a) controlling the HAR to vary the laser transmission period ($T_{P}$) of a laser diode (LD) depending on the vehicle’s speed and (b) reducing the static power consumption using a sleep mode, depending on the surrounding environment. The proposed LiDAR sensor with the HAR control algorithm reduces the power consumption of the LD by 6.92% to 32.43% depending on the vehicle’s speed, compared to the maximum number of laser transmissions ($N_{x.max}$). The sleep mode with a surrounding environment-sensing algorithm reduces the power consumption by 61.09%. The algorithm of the proposed LiDAR sensor was tested on a commercial processor chip, and the integrated processor was designed as an IC using the Global Foundries 55 nm CMOS process.

## 1. Introduction

**Figure 1:**
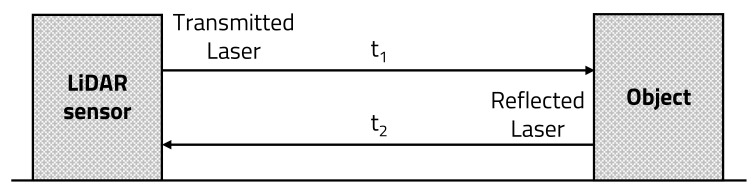
Time-of-flight (ToF) of laser.

With the rapid development of automotive technology, the types and quantities of sensors mounted on automobiles are gradually increasing, and the quantitative and qualitative improvement of sensors provides safety and convenience for drivers and passengers [1,2,3]. Therefore, because recently developed autonomous vehicles use a large number of various sensors, research has focused on the safety of and convenience for drivers, as well as on fusion signal processing among a number of used sensors [4,5]. Among the sensors that provide safety and convenience, light detection and ranging (LiDAR) sensors are the most essential for autonomous vehicles because they measure the distance between a vehicle and an object and recognize the object [6,7]. As shown in Figure 1, a typical LiDAR sensor using a laser in 905 nm of the near-infrared ray (NIR) region uses technology to convert the time-of-flight (ToF), which is the time difference between the transmission of the laser ($t_{1}$) and its reflection from an object back to the sensor ($t_{2}$) [8,9,10,11].

**Figure 2:**
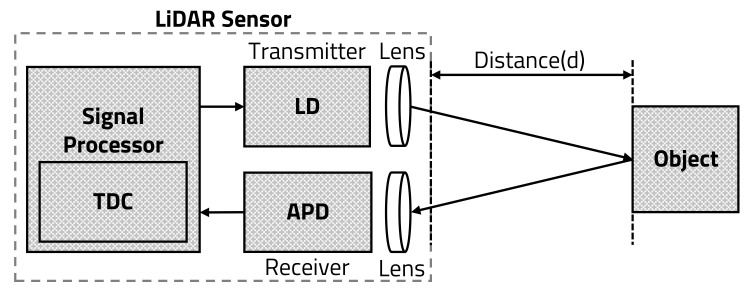
Structure and operation of conventional light detection and ranging (LiDAR) sensor.

A LiDAR sensor, which detects the exact distance from an object by transmitting a laser, consists of a laser diode (LD), an avalanche photo diode (APD), a time-to-digital converter (TDC), and signal processing units, as shown in Figure 2 [12].

The LD transmits the laser, which is focused through a light transmitting lens. The laser transmitted from the LD is reflected back from the object and received by the APD through the light-receiving lens. The TDC measures the difference between the time the LD transmits the laser and the time the APD receives it, and then converts the difference to the ToF. The signal-processing unit, denoted by a microprocessor (MP), receives the ToF from the TDC and calculates the distance between the LiDAR sensor and the object.

The laser of the LiDAR sensor can be classified into a pulsed, amplitude-modulated continuous-wave (AMCW), and frequency-modulated continuous-wave (FMCW) according to the transmission principle. The pulsed is a method of transmitting a laser with an instantaneous peak power using a short pulse of several nanoseconds. Because an intensity of an instantaneous laser is strong, it is used for long distance measurement. The AMCW is a method of transmitting a continuous laser. The distance is measured by comparing phases of the transmitted and backscattered detected waves. Because it measures a phase shift of the transmitted and received laser, it is not suitable for an accurate distance and long distance measurement [13]. The FMCW is a method that compensates for AMCW’s incorrect distance measurement. It transmits the laser continuously, but the frequency changes with time. The accurate distance measurement is possible because the laser transmission time can be known through the frequency of the received signal. However, it is structurally more complex than the AMCW and is not suitable for the long distance measurement because the frequency changes due to the Doppler effect [14].

The types of LiDAR sensors that measure distance are categorized into scanning sensors, microelectromechanical system (MEMS) sensors, flash sensors, and optical phased array (OPA) sensors. Scanning and MEMS sensors use mirrors to spread the laser to detect multiple directions. Scanning sensors use a mirror rotated by a motor to change the angle at which the laser is reflected and transmitted [15,16]. MEMS sensors use the mirror to transmit the laser in the same way as scanning sensors, but the mirror is operated vertically, horizontally, or in all directions [17,18,19]. Flash sensors transmit one large-area laser, such as with a digital camera, and receive the laser reflected by the object in an array sensor, which is expressed in one frame [20,21]. OPA sensors use a method in which an optical phase modulator controls a phase of the laser through the lens and transmits the laser in various directions [22,23].

In this paper, among the various types of LiDAR sensors, we use a scanning LiDAR sensor with a pulsed laser, which is best suited for autonomous vehicles requiring a wide field of view.

This paper is organized as follows. Section 2 briefly discusses the related works. Then, the problems of conventional LiDAR sensors and the method by which the proposed LiDAR sensors reduce power consumption are described in Section 3. In Section 4, the LiDAR sensor with reduced power consumption is verified through experiments. Finally, Section 5 contains the conclusion.

## 2. Related Works

As numerous high-tech devices are developed in various fields, miniaturization is pursued for convenience and portability. In addition, low-power issues are being discussed to enable the operation of small batteries for portability. To satisfy these requirements, the structure of the signal processing hardware has been modified in the past to reduce power consumption [24,25]. Recently, the trend is to reduce power consumption with a low-power operation algorithm and efficient data processing [26,27,28].

Radio detection and ranging (RADAR) sensors, similar to LiDAR sensors, have been applied in various fields for a long time, but have recently been introduced in vehicles [4]. As RADAR sensors develop in sophistication, the research on low-power consumption of RADAR sensors continues [29,30]. However, because the research period of multi-channel LiDAR sensors has not been relatively long, judging by the technology trend, the research and development of an improved sensing distance, a point-cloud signal-processing method, and accuracy and recognition are being conducted [31,32,33,34]. Because the research of a multi-channel LiDAR sensor is focused on improving performance, the research on low-power consumption for vehicles has been insufficient.

Currently, the conventional LiDAR sensors on the market have the disadvantage of inefficient power consumption because of the constant operation of a vertical angular resolution (VAR) and horizontal angular resolution (HAR) fixed. In particular, because the fatal disadvantage of the LiDAR sensor causes vulnerabilities in autonomous vehicles equipped with multiple sensors and electric vehicles using batteries as a power source, it is necessary to improve the operation method to achieve efficient power consumption [1,2]. A solution to the power consumption should be prepared according to the advanced performance of the LiDAR sensor. A simple method of reducing the power consumption involves modifying the hardware of the LiDAR sensor by focusing on an algorithm that changes the fixed accuracy of the conventional LiDAR sensor according to the condition.

The LiDAR sensor controls and changes the laser transmission period ($T_{P}$), which is the HAR, according to the vehicle’s speed and surrounding environment. Because the number of laser transmissions ($N_{x}$) varies proportionally according to $T_{P}$, we propose an algorithm that can efficiently consume power according to the variable $T_{P}$ [35].

## 3. Characteristics of Multi-Channel Scanning LiDAR Sensors

### 3.1. Conventional Multi-Channel Scanning LiDAR Sensors

**Figure 3:**
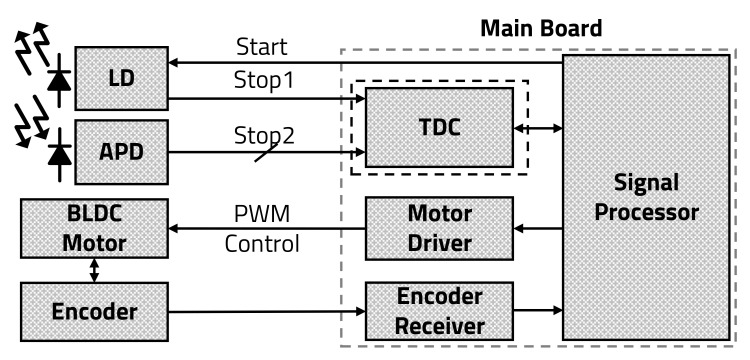
Block diagram of multi-channel scanning LiDAR sensor.

As mentioned in the introduction, a multi-channel scanning LiDAR sensor is configured with a brushless direct current (BLDC) motor, which rotates the mirror, and an encoder within the structure of the typical LiDAR sensor, as shown in Figure 3. In addition, the multi-channel scanning LiDAR sensor uses a multi-channel APD, which determines a vertical field of view (VFoV). The encoder extracts the angle information of the BLDC motor, which rotates the mirror, and transmits it to the microprocessor (MP).

The MP calculates the motor’s rotation angle using the angle data received from the encoder and controls the duty cycle of a pulse width modulation (PWM) signal with the motor driver. When the mirror’s angle is in the detection range, the MP triggers a START signal to the LD. The LD, which receives the START signal from the MP, transmits a STOP1 signal to the TDC at the same time as the laser transmission. The TDC that received the STOP1 signal from the LD counts using an internal timer (TMR) until STOP2 signals from each channel of the APD come in. Each channel of the APD receives the laser reflected by the object and sends the STOP2 signal to stop the TDC from counting. The TDC transmits its count values from the STOP1 signal to the multi-channel STOP2 signal and then to the MP. After receiving the count value from the TDC, the MP converts the distance value by applying the speed of light: $C=3*10^{8}$ m/s. The LiDAR sensor repeats these operations by the value of a horizontal field of view (HFoV) at different mirror angles.

#### 3.1.1. Time-Domain of TDC Operations

**Figure 4:**
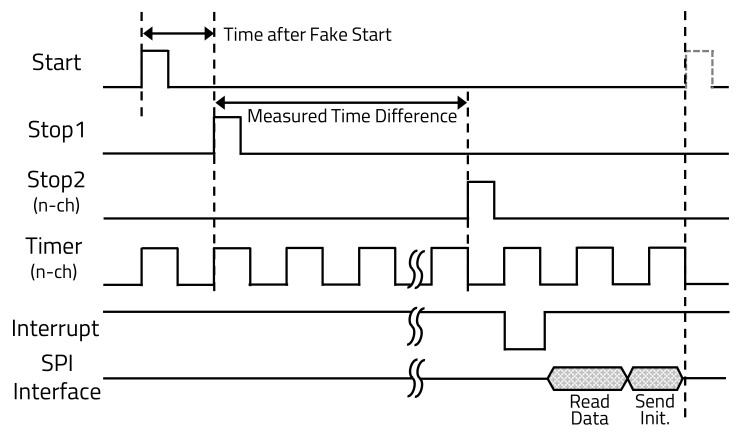
Time domain of time-to-digital converter (TDC).

Figure 4 shows the TDC operations process of each input signal to the TDC during the operation time and the data transmitted to the MP after signal processing [36,37,38].

When the MP sends the START signal to the LD and TDC, the TDC that receives the START signal waits for the STOP1 signal from the LD. The LD that received the START signal from the MP transmits the laser and sends a STOP1 signal to the TDC at the same time. When the laser reflected from the object reaches each channel of the APD, the STOP2 signals are transmitted to the TDC. The TDC’s TMR counts from the input time of the STOP1 signal until the STOP2 signal is received by each channel of the APD. When the TMR finishes counting because all channels received STOP2 signals, the TDC sends an interrupt signal, which is the signal that reception is complete for all channels, and the ToF data of each channel are transmitted to the MP using a serial peripheral interface (SPI) communication.

#### 3.1.2. Power Consumption of LiDAR Sensor

**Figure 5:**
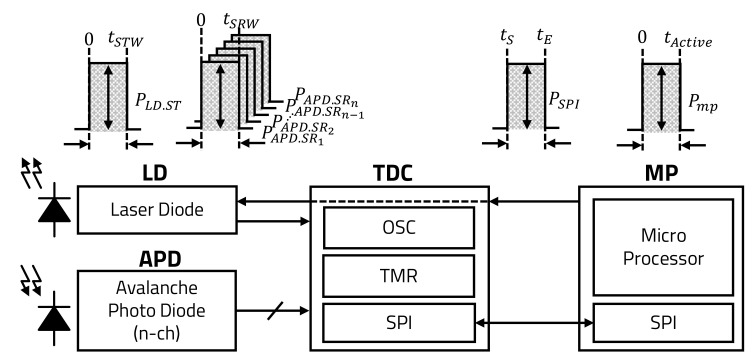
Power consumption of LiDAR sensor.

Figure 5 shows the overall block diagram of the LiDAR sensor when the LD of the LiDAR sensor transmits the pulsed laser once. When the LD transmits a single pulsed laser, the multi-channel APD receives the laser reflected from the object, and the received signal of the APD is transmitted and processed by the TDC and MP.

The energy consumption of one pulsed laser pulse transmitted from LD, as denoted by $E_{LD}$, is expressed as shown in Equation (1). The LD consumes dynamic power for several nanoseconds, as denoted by $P_{LD.ST}$, from the time 0, when the laser transmission starts, to the time $t_{STW}$, when the transmission ends. $E_{LD}$, which is the energy of one pulsed laser, is expressed as the integration of $P_{LD.ST}$ from 0 to $t_{STW}$.

(1) $$E_{LD}=\int_{0}^{t_{STW}}P_{LD.ST}\left(t\right)dt$$

Each channel of the APD statically consumes power, as denoted by $P_{APD.SR_{i}}$, to recognize the laser reflected by the object. For one channel of the APD, the power consumption is the integration of $P_{APD.SR_{i}}$ from 0, when the reception is started, to $t_{SRW}$ at which the reception of each channel is ended. Generally, the number of the APD’s channels is determined by *n* cells. The total energy consumption of the APD’s *n* channels, as denoted by $E_{APD}$, is expressed as the sum of the power consumption used in each channel of the APD, as shown in Equation (2).

(2) $$E_{APD}=\sum_{i=1}^{n}\left[\int_{0}^{t_{SRW}}P_{APD.SR_{i}}\left(t\right)dt\right]$$

**Figure 6:**
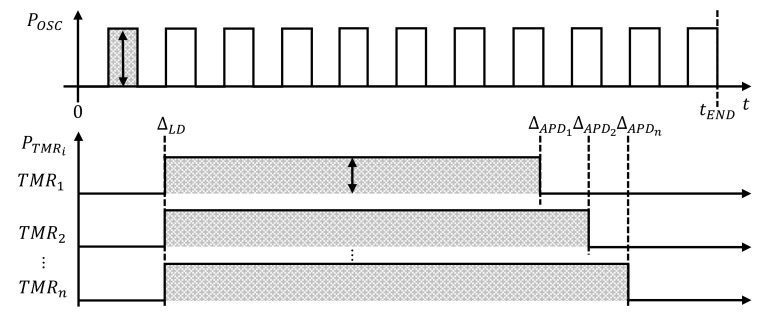
Power consumption of TDC.

The TDC has an oscillator (OSC) that consumes power, as denoted by $P_{OSC}$, during each high-output period to count up each TMR. The energy consumption of the OSC is an integration of $P_{OSC}$ from 0 to $t_{END}$ when a group of data is processed. Each TMR consumes its static power, as denoted by $P_{TMR_{i}}$, when it operates from the time it receives the LD signal, as denoted by $\Delta_{LD}$, to the time when each channel receives the APD signal, denoted by $\Delta_{APD_{i}}$, as shown in Figure 6. $E_{TDC}$ denotes the sum of the oscillator’s energy consumption and the integration of $P_{TMR_{i}}$’s power from $\Delta_{LD}$ to $\Delta_{APD_{i}}$ of each TMR, as shown in Equation (3).

(3) $$E_{TDC}=\int_{0}^{t_{END}}P_{OSC}\left(t\right)dt+\sum_{i=1}^{n}\left[\int_{\Delta_{LD}}^{\Delta_{APD_{i}}}P_{TMR_{i}}\left(t\right)dt\right]$$

The operating power of SPI communication, which is implemented for faster communication at the hardware level and widely used for data transmission between sensors, is also consumed during such data communication. As shown in Equation (4), the energy consumed by the SPI communication between the TDC and the MP, as denoted by $E_{TP}$, is expressed as the integration of the SPI’s power consumption, as denoted by $P_{SPI}$, from the time $t_{S}$ when the transmission starts to the time $t_{E}$ when the transmission ends.

(4) $$E_{TP}=\int_{t_{S}}^{t_{E}}P_{SPI}\left(t\right)dt$$

When the MP receives data from the TDC via the SPI communication, the MP’s power consumption, as denoted by $P_{MP}$, is represented by Equation (5). $E_{P}$ denotes the integration of the MP’s instantaneous power consumption, as denoted by $P_{MP}$, from 0, when the data are transmitted from the TDC, to $t_{Active}$, when the MP completes signal processing.

(5) $$E_{P}=\int_{0}^{t_{Active}}P_{MP}\left(t\right)dt$$

The LiDAR sensor’s total energy consumption, as denoted by $E_{Total}$, is expressed as shown in Equation (6).

(6) $$E_{Total}=E_{LD}+E_{APD}+E_{TDC}+E_{TP}+E_{P}$$

If the LiDAR sensor, which periodically transmits the laser with the certain HAR, detects objects, it consumes a constant energy as much as $E_{Total}$. On the other hand, if a laser transmission period of the conventional LiDAR is changed, $E_{Total}$ will also be changed. Because the accuracy changes when $T_{P}$ of the LD is varied, the LiDAR sensor is correlated with $E_{APD}$, $E_{TDC}$, and $E_{P}$. Due to the change of $E_{LD}$, the total energy consumption $E_{Total}$ can be reduced.

**Figure 7:**
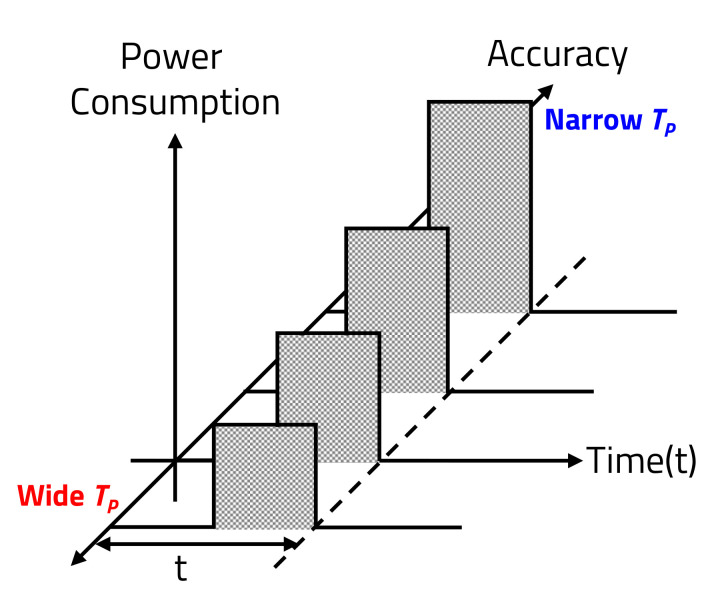
Relation of power consumption and accuracy.

$T_{P}$ refers to the time interval for transmitting the laser at the HAR intervals. As shown in Figure 7, if the HAR is increased by narrowing $T_{P}$, the LiDAR sensor’s power consumption increases as the accuracy of the object detection increases.

In addition, if the HAR is decreased by widening $T_{P}$, the power consumption decreases as the accuracy decreases. Therefore, because $T_{P}$ of the LD, which determines the HAR, and the accuracy of the object detection are proportional, power consumption increases as $T_{P}$ narrows for the same period of time.

### 3.2. Proposed LiDAR Sensor

In this paper, we propose methods to efficiently reduce the sensor’s power consumption by varying the HAR of the 16-channel scanning LiDAR sensor. The proposed LiDAR sensor is designed and manufactured for vehicles and has the specifications as shown in Table 1. As mentioned in Section 1, the proposed LiDAR sensor detects objects up to 150 m using a NIR 905 nm laser based on pulsed ToF technology. The proposed LiDAR communicates an external device using BroadR-Reach (BRR), which is an Ethernet for vehicle, and additionally has a controller area network (CAN) communication to enable connection with the vehicle’s engine control unit (ECU). In addition, the proposed LiDAR sensor is designed to operate at 12 V, which is an operation voltage for vehicles, up to 36 V. Therefore, it can be driven at even more than 24 V, which is a power supply for a bus.

**Figure 8:**
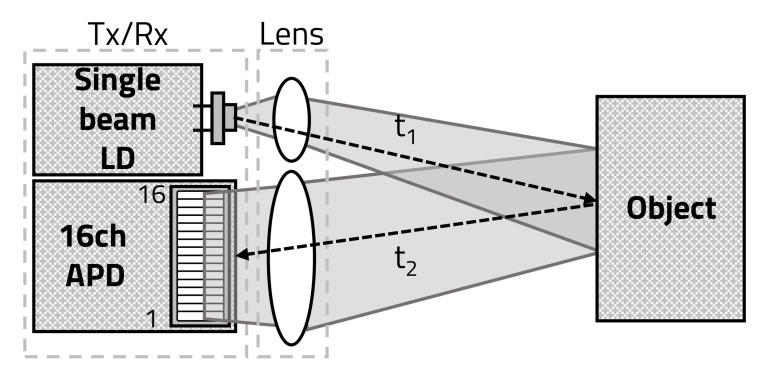
Structure of propoesd LiDAR sensor’s sensing.

The LD of the proposed LiDAR sensor transmits a single-beam laser, which is the VFoV of 9.6${}^{{}^{\circ}}$. The pulse width of the laser is 10 ns and frequency is about 86 kHz, when the HAR is 0.25${}^{{}^{\circ}}$. A single 16-channel APD, which consist of 16 sensing cells, receives the laser reflected by the object into almost similar frequency as shown in Figure 8.

Therefore, the proposed LiDAR sensor’s VAR is determined by the APD’s 16 sensing cells as 0.6${}^{{}^{\circ}}$. The LiDAR sensor’s HAR is determined by the number of the laser transmissions within the HFoV. When the motor is rotated at 30 Hz (=30 cycles), the LiDAR sensor takes about 33.33 ms to rotate at one cycle (360${}^{{}^{\circ}}$), and a sensing angle of 145${}^{{}^{\circ}}$ takes about 13.43 ms. Therefore, $N_{x}$ is determined as shown in Table 2 by the HAR as taking 13.43 ms.

**Figure 9:**
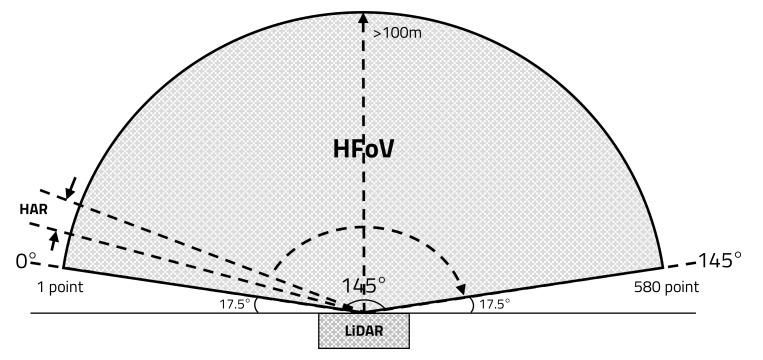
Horizontal field of view (HFoV) of LiDAR sensor.

As shown in Figure 9, the proposed LiDAR sensor that can measure more than 100 m uses the reflection mirror mounted on the BLDC motor, which rotates to 30 Hz and transmits a laser 580 times at 0.25${}^{{}^{\circ}}$ intervals—a basic HAR—within the HFoV of 145${}^{{}^{\circ}}$.

**Figure 10:**
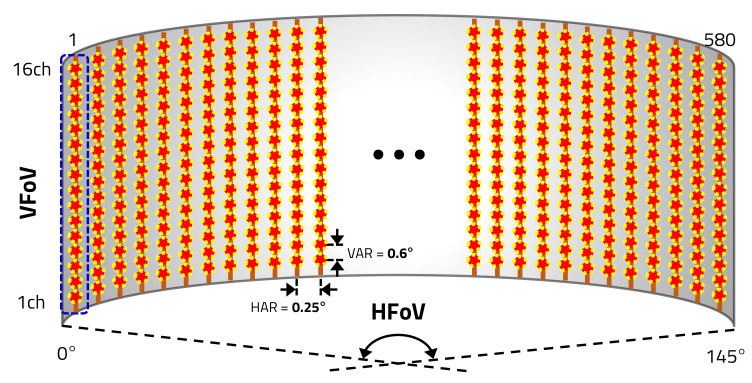
Point cloud of LiDAR sensor.

As shown in Figure 10, the 16-channel LiDAR sensor, which detects the object, implements one frame using the VFoV of 9.6${}^{{}^{\circ}}$ and the HFoV of 145${}^{{}^{\circ}}$.

With these specifications, we propose methods to efficiently reduce the power consumption of the LiDAR sensor from two perspectives: the vehicle’s speed and the sensor’s surrounding environment. In terms of the vehicle’s speed, the LiDAR sensor controls the HAR to reduce the dynamic power consumption. In terms of the sensor’s surroundings, the LiDAR sensor goes into sleep mode to minimize the static power consumption.

#### 3.2.1. Speed Detection-Based LiDAR Sensor Control

A LiDAR sensor with the same HAR, $T_{P}$, detects the object more accurately at a short distance than at a long distance because of the angle at which the laser is transmitted. Therefore, when considering the distance to the object, if the laser’s HAR is low, the LiDAR sensor can be used for short-range object detection, and the higher the laser’s HAR, the more suitable it will be for detecting long-range objects. We designed the LiDAR sensor so that the LD’s $T_{P}$ depends on the vehicle’s speed, as depicted in Algorithm 1. When the vehicle’s speed is faster than 100 km/h, the LiDAR sensor uses the maximum accuracy to detect distant objects at high speed. However, when the vehicle’s speed is slower than 100 km/h, $N_{x}$ is reduced for low-power operation. As shown in Table 3, when the vehicle’s speed is faster than 100 km/h, the LiDAR sensor transmits the laser 580 times to the maximum number of laser transmissions ($N_{x.max}$) with 0.25${}^{{}^{\circ}}$ HAR. When the vehicle is driving at medium-high speed (80–100 km/h), the LiDAR sensor transmits the laser 483 times at 0.3${}^{{}^{\circ}}$ HAR, so our method reduces $N_{x}$ by 16.73%. $N_{x}$ is decreased by 28.62% with 414 laser transmissions and 0.35${}^{{}^{\circ}}$ HAR, when the vehicle’s speed is medium (60–80 km/h). For medium-low speeds (40–60 km/h), the LD transmits the laser 362 times, which reduce $N_{x}$ by 37.56%, and the HAR is 0.4${}^{{}^{\circ}}$. If the vehicle’s speed is slower than 40 km/h, then the minimum number of laser transmissions ($N_{x.min}$), 322, can decrease $N_{x}$ by 44.48% with a 0.45${}^{{}^{\circ}}$ HAR.

**Figure 11:**
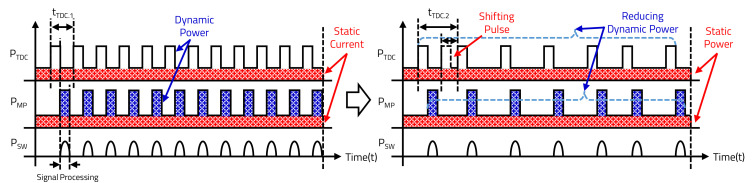
Power consumption of speed detection-based LiDAR sensor.

Figure 11 shows the power consumption of the TDC and MP. The static power denoted as an area of the static current consumption, which is consumed continuously when the sensor is activated, is the same regardless of the power consumption control. However, the LD’s $N_{x}$ decreases according to the vehicle’s speed, and the dynamic power denoted as an area of the dynamic current consumption, which is the sum of the power required to transmit the data at the TDC and the power required to process the data at the MP, is decreased.
**Algorithm 1:** Speed detection-based accuracy control.
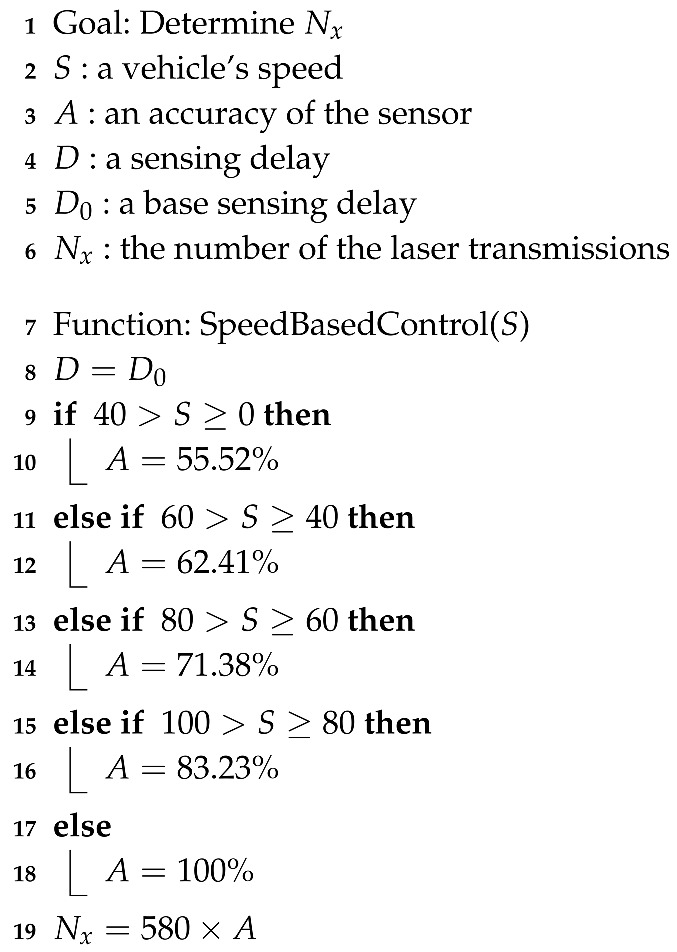


#### 3.2.2. Environment Sensing-Based LiDAR Sensor Control

**Figure 12:**
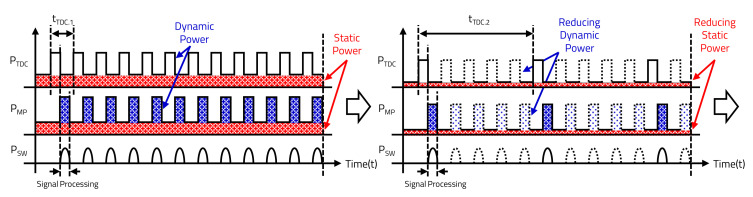
Power consumption of environment sensing-based LiDAR sensor.

**Figure 13:**
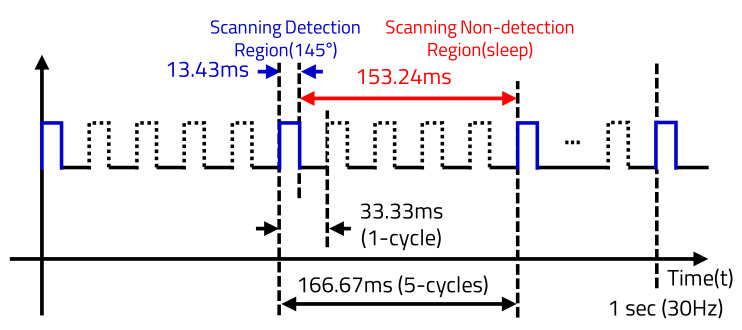
Time domain of sleep mode.

The LiDAR sensor does not need to detect the surroundings with high HAR when the vehicle is stopped or when objects are not detected at very long distances. Based on the environmental detection results, the MP reduces the power consumption by entering sleep mode, which commands the LiDAR sensor to sleep. As shown in Figure 12, the LD of the LiDAR sensor transmits the laser at every cycle in the speed detection mode, and $T_{P}$ varies depending on the vehicle’s speed. However, because the LD transmits only one cycle out of five cycles in sleep mode, not only is $T_{P}$ variable, but the static power consumption is also reduced. As shown in Figure 13, the time to detect only 145${}^{{}^{\circ}}$ out of the one cycle (360${}^{{}^{\circ}}$) is 13.43 ms, and the time of the non-detection region, in which LD is not transmitted, is 153.24 ms. In addition, the LiDAR sensor in sleep mode transmits 290 times with 0.5${}^{{}^{\circ}}$ HAR, which is half at $N_{x.max}$ when the vehicle’s speed is faster than 100 km/h, as shown in Table 4.

The environment sensing-based LiDAR sensor reduces the power consumption in two cases: when the vehicle is moving or not moving. When the vehicle is driving, the LiDAR sensor operates based on the method represented in Section 3.2.1, in which $T_{P}$ is controlled by the vehicle’s speed. However, if the sensor does not detect any objects for a certain time period, the LiDAR sensor will determine that there are no objects around the vehicle, and it will enter sleep mode to minimize the detection speed and power consumption. The sensor will detect the surroundings when the vehicle stops or waits for a traffic signal. The LiDAR sensor enters sleep mode when the surrounding objects have not moved for a certain time period. In sleep mode, the LiDAR sensor regularly detects the surrounding environment every five cycles, and maintains the sleep mode if no object is detected. If the environment, which the sensor detects periodically, is changed or the vehicle starts to move, the LiDAR sensor returns to the active mode and detects objects with normal $T_{P}$, as depicted in Algorithm 2.
**Algorithm 2:** Environment sensing-based accuracy control.
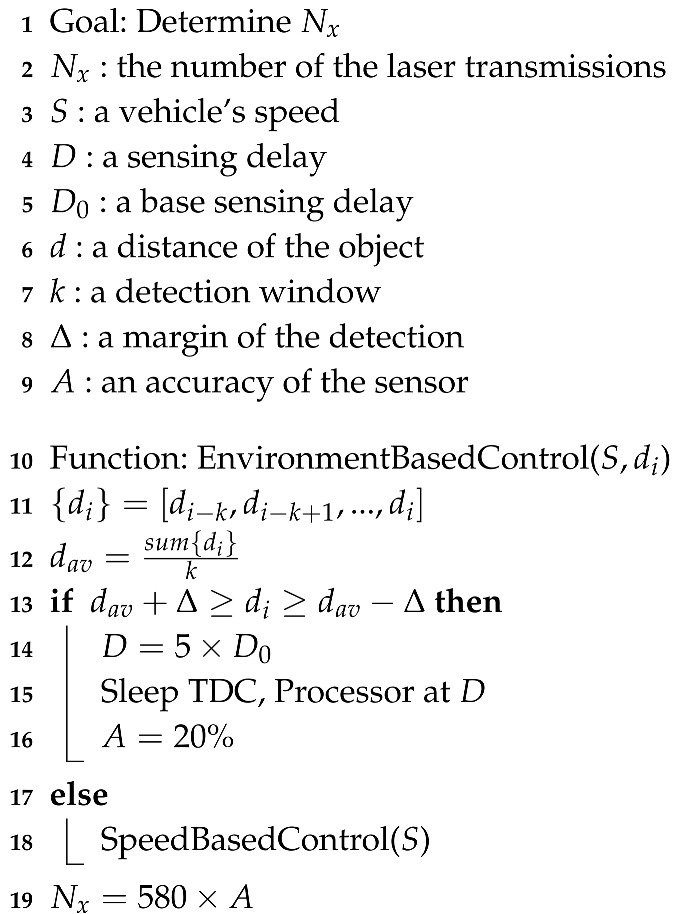


## 4. Implementation and Experiment

**Figure 14:**
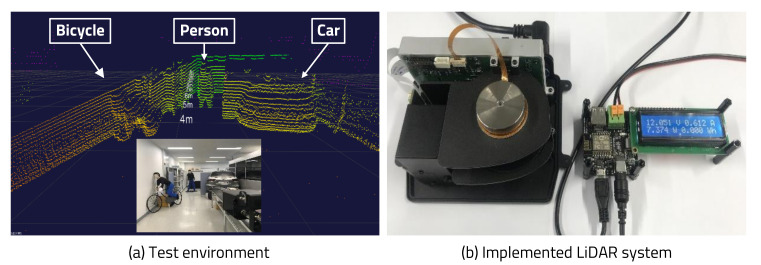
Test environment and implemented LiDAR system. (**a**) Test environment for LiDAR evaluation (bicycle, person, and car are detected). (**b**) Power consumption measurement setup for evaluating implemented LiDAR system.

For an experiment with the proposed algorithm, we designed private hardware based on a Zynq7020 processor (San Jose, CA, USA) and manufactured a LiDAR sensor module. To verify the LiDAR sensor, a test environment through the LiDAR sensor and viewer program was configured as shown in Figure 14a. The dedicated viewer program to check the operation status of the LiDAR sensor was implemented using the open source edition of the Qt framework and the point cloud library (PCL). In addition, the LiDAR sensor’s power consumption was measured using a LiDAR sensor and a smart power debugger, a verification system, as shown in Figure 14b.

The experiment was divided into two types, and the simulation results of the produced chip are shown based on the experimental results. First, the resulting change in $T_{P}$ was compared after the experiment with variable $T_{P}$ according to the variable HAR of the LiDAR sensor’s operation. Second, in order to confirm the effect of the proposed LiDAR sensor’s algorithm, the variable HAR and the sleep mode are applied, and the LiDAR sensor’s power consumption was measured to verify that the power consumption varied according to the HAR.

### 4.1. Power-Consumption Measurement of LiDAR Sensor

**Figure 15:**
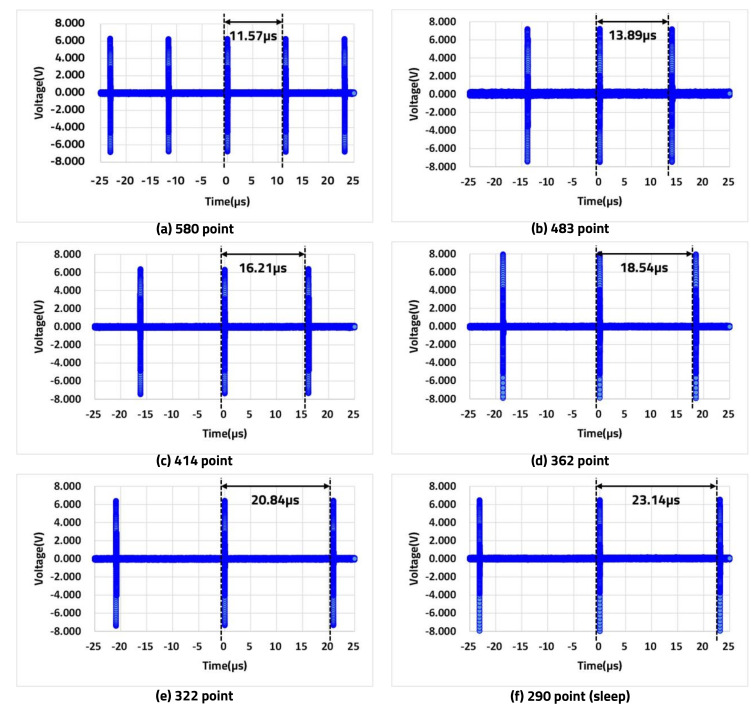
Comparison of $T_{P}$. (**a**) 580 laser transmissions at 11.57 μ s intervals. (**b**) 483 laser transmissions at 13.89 μ s intervals. (**c**) 414 laser transmissions at 16.21 μ s intervals. (**d**) 362 laser transmissions at 18.54 μ s intervals. (**e**) 322 laser transmissions at 20.84 μ s intervals. (**f**) 290 laser transmissions at 23.14 μ s intervals.

The following experiment results confirm the variation of $T_{P}$ according to the variable HAR of the LiDAR sensor. Based on Table 2 as shown in Section 3.1.2, when the HAR of $N_{x.max}$ (580 times) is 0.25${}^{{}^{\circ}}$, we confirmed that the LD transmits the laser every 11.57 μ s through an oscilloscope. In the same way, data were extracted for $T_{P}$ from 0.3${}^{{}^{\circ}}$ to 0.5${}^{{}^{\circ}}$ in sleep mode, as shown in Figure 15. The results confirmed that $T_{P}$ increased as the HAR decreased.

**Figure 16:**
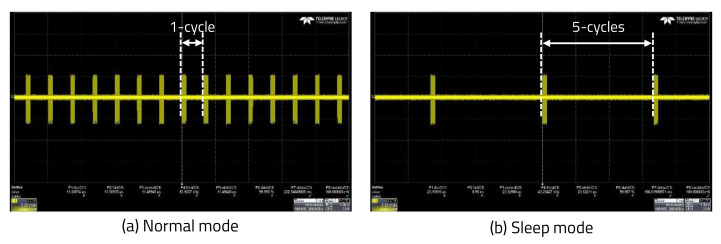
Comparison of $T_{P}$ in normal and sleep mode. (**a**) Laser transmissions of Nx every cycle in normal mode. (**b**) Laser transmissions of 290 times for only 1-cycle in 5-cycles in sleep mode.

Additionally, the LiDAR sensor’s HAR was varied by transmitting the laser every rotation, but in sleep mode, the laser was transmitted only once every five rotations to further reduce the power consumption in the environment sensing-based accuracy control mode. $T_{P}$ was confirmed using an oscilloscope, as shown in Figure 16.

**Figure 17:**
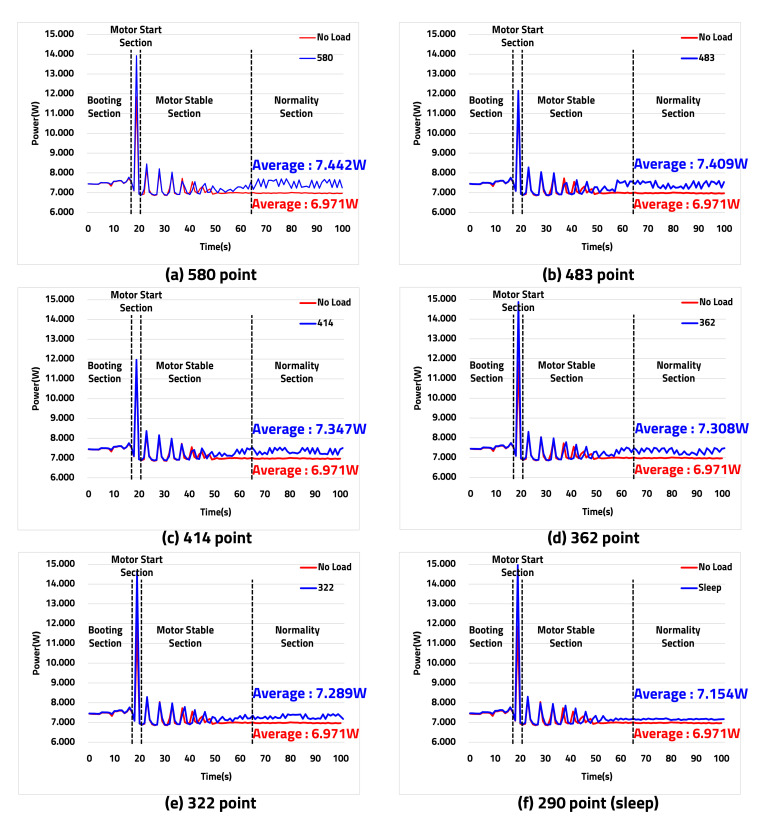
Comparison of power consumption depending on $N_{x}$. (**a**) Average power consumption graph of 7.442 W in 580 laser transmissions. (**b**) Average power consumption graph of 7.409 W in 483 laser transmissions. (**c**) Average power consumption graph of 7.347 W in 414 laser transmissions. (**d**) Average power consumption graph of 7.308 W in 362 laser transmissions. (**e**) Average power consumption graph of 7.289 W in 322 laser transmissions. (**f**) Average power consumption graph of 7.154 W in 290 laser transmissions.

Previously, the output of $T_{P}$ was confirmed through experiments, and the LiDAR sensor’s power consumption according to $T_{P}$ was measured as shown in Figure 17. The measured results are divided into boot, motor (start, stabilization), and normality sections. The data for the actual boot and motor sections were considered invalid because it is the power use section before normality section. Only valid data of the normality section were extracted and expressed, as shown in Table 5.

In a red waveform of the normality section, an average current (AC) of 0.578 A and an average power consumption (APC) of 6.971 W were obtained using the LiDAR sensor’s power consumption in the no-load state without driving the LD. The blue waveform shows the APC of each from 7.442 W to 7.154 W, from $N_{x.max}$ to $N_{x.min}$, with the LD mounted and in sleep mode.

As described above, the APC is reduced when varying $N_{x}$ depending on the vehicle’s speed. When the vehicle is driving at low speed, the power consumption is reduced because the HAR, controlled by $N_{x}$, is lowered by the algorithm of the speed detection-based LiDAR sensor control. In particular, the sleep mode is reduced by 3.86% of the power consumption as compared to $N_{x.max}$. The power consumption can be improved by varying the HAR according to the vehicle’s speed. In addition, from the LD’s point of view, while the APC of the LiDAR sensor in the no-load state is 6.971 W, the power difference for the LD excluding only the no-load APC from $N_{x.max}$ to $N_{x.min}$ is expressed as shown in Table 6. When $N_{x}$ is 580 times, the difference in the average current (DAC) is 39.0 mA, and the difference in the average power consumption (DAPC) obtained by subtracting the APC at the no-load state, excluding from the APC at $N_{x.max}$, is 470.4 mW.

In the sleep mode with $N_{x.min}$, the DAPC is 183.0 mW, which is 61.09% lower compared to $N_{x.max}$. In this case, the LD’s power reduction rate (PRR) is changed step by step depending on the vehicle’s speed. However, when $N_{x}$ is 322 times, the PRR of sleep mode is approximately 1.8 times different from the PRR of 32.43%. As a result, the PRR in sleep mode is increased because it does not transmit the laser every cycle but only on one cycle out of five cycles. Therefore, the variable and HAR control of $T_{P}$ being dependent on the vehicle’s speed was verified by applying the speed detection algorithm, and the reduced power consumption of the LiDAR sensor was confirmed through the experimental results by additionally applying the environment-sensing algorithm.

The proposed LiDAR sensor’s algorithm clearly has a risk factor of four cycles (153.24 ms) that does not detect an object in sleep mode during the environmental-sensing control. This issue is considered to be negligible because it immediately switches to active mode when surrounding conditions change in sleep mode when the vehicle is stop. In the future, further work on an adaptive mode that immediately exits sleep mode when an event occurs through dynamic control of sleep mode in conjunction with other sensors (images, RADAR sensors and etc.) of the autonomous vehicle is needed.

### 4.2. Chip Designed for the LiDAR Sensor

**Figure 18:**
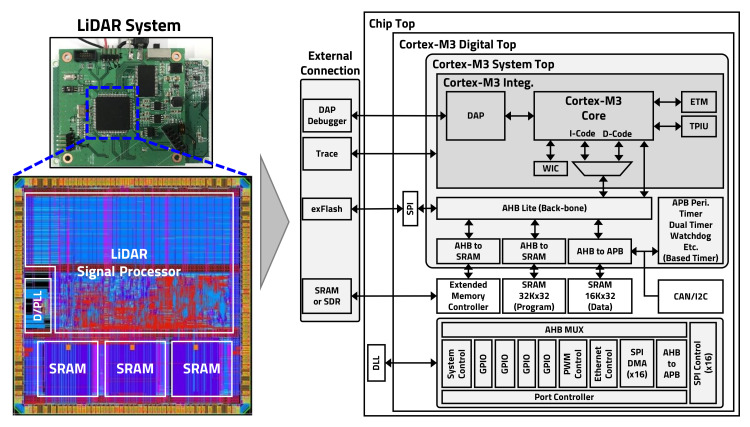
Designed microprocessor (MP) chip.

A designed chip was manufactured as shown in Figure 18 using the Global Foundries (Santa Clara, CA, USA) 55 nm process library, and it was implemented using approximately 540,000 gates, including an ARM core.

The chip is a MP with a built-in ARM Cortex-M3 (Cambridge, United Kingdom) that has 128 KB of program memory and 64 KB of data memory. In addition, we designed the chip to use external expansion memory in case the internal memory capacity is insufficient, which can be confirmed by the block diagram shown in Figure 18. A DLL designed on the chip can receive 4–24 MHz of clock from the outside and set the internal operation clock up to 160 MHz. The CPU fetches the program from the internal SRAM. The programming method was designed in two modes. The first mode directly programs the code to SRAM through I2C or debugger, and the second mode is executed by copying the program to SRAM by programming to external eFlash. An interface of the TDC is designed to support three types of TDC. The input can read data using 16 one-channel TDCs through SPI or read data using one 16-channel TDC. In addition, data can be received via parallel transmission from the CMOS image sensor. The Ethernet port was designed with the SPI. An I2C slave controller was used to control a hardware register, and two modes were applied: a mode for accessing registers directly and one for access by communicating directly with the CPU. In addition, a timing control block for controlling the PWM was configured for the LD’s $T_{P}$ and to rotate the BLDC motor. Different timings could be controlled so that each block could operate independently. The PWM signal can generate desired waveforms by controlling the start latency, pulse width, pulse period, number of generated signals, and pulse hold. As described above, a dedicated chip was designed and manufactured for the LiDAR sensor, and the proposed algorithm will be applied to the manufactured chip for testing.

## 5. Conclusions

This paper proposes an algorithm to improve the inefficient constant operation method of conventional LiDAR sensors. Conventional LiDAR sensors inefficiently consume the operating power because they do not consider the vehicle’s speed or the surrounding conditions. The proposed LiDAR sensor is implemented with software and hardware using the proposed algorithm to reduce the power consumption by varying the accuracy of a LiDAR sensor to be applied to electric and autonomous vehicles with limited power consumption. The proposed power-control algorithm operates according to two viewpoints: the vehicle’s speed and the surrounding environment. First, the LiDAR sensor controls the laser’s HAR based on the vehicle’s speed to reduce the dynamic power consumption. Second, when the surrounding environment does not change for a certain time period, the proposed LiDAR sensor reduces the static power consumption by minimizing unnecessary detection through sleep mode. When the HAR was operated at 55.52% of $N_{x.max}$ at low speed using the proposed LiDAR sensor, the LD’s APC was reduced by 32.43%, and when driving only one cycle per five cycles, the APC of the LD was reduced by 61.09%, and the HAR in sleep mode was reduced to 50% of $N_{x.max}$. The proposed LIDAR sensor applied the algorithm to control the HAR, which is $T_{P}$, according to the vehicle’s speed, and successfully tested the LiDAR sensor using a verification system, which efficiently reduced the power consumption. Therefore, the proposed LiDAR sensor can flexibly cope with various vehicle environments and improve the efficiency of the operations with the applied algorithm. In addition, the manufactured chip for the LiDAR sensor uses the Global Foundries 55 nm CMOS process, and the designed processor area is 1,383,647 μ m ${}^{2}$ and is about 540,000 gates. The chip has an ARM Cortex-M3 processor, and the designed processor will be tested using the proposed algorithm based on embedded software. The chip is expected to be completely redesigned with an on-chip MP and embedded software to optimize the power consumption as the environmental changes. In this paper, only the LD’s power consumption of the LiDAR sensor was controlled by applying an algorithm that changes the HAR according to the vehicle’s speed and detects the environment. Future research will be conducted to reduce the power consumption more efficiently by controlling parts other than the LD. It was determined that the proposed LiDAR sensor can be applied not only to the automotive industry but also to various fields, including the robot and drone industries, and that the power consumption algorithm can be applied in suitable ways for the field. 

## Figures and Tables

**Table 1:** Proposed LiDAR sensor specifications.

Model	Features	Specification
16 ch LiDAR	Channels	16 ch (Parallel layers)
	Technology	Pulsed ToF
	Source	NIR 905 nm
	HFoV	145${}^{{}^{\circ}}$
	VFoV	9.6${}^{{}^{\circ}}$
	Scanning frequency	30 Hz
	Angular resolution	H : 0.25${}^{{}^{\circ}}$ / V : 0.6${}^{{}^{\circ}}$
	Range	≤150
	Interface	BroadR–Reach Ethernet / CAN / RS–232
	Viewer	Self-development using Qt
	Operation voltage	10–36 VDC
	Power Consumption	≤7.5 W

**Table 2:** $N_{x}$ depending on horizontal angular resolution (HAR).

HFoV (${}^{{}^{\circ}}$)	HAR (${}^{{}^{\circ}}$)	$N_{x}$	Term (μ s)
145	0.25	580	11.57
	0.3	483	13.89
	0.35	414	16.21
	0.4	362	18.54
	0.45	322	20.84
	0.5	290	23.14

**Table 3:** $N_{x}$ depending on vehicle’s speed.

Speed (km/h)	HAR (${}^{{}^{\circ}}$)	$N_{x}$	$N_{x}$ Rate (%)
S ≥ 100	0.25 (–)	580 (–)	100 (–)
100 > S ≥ 80	0.3 (+0.05)	483 (–97)	83.23 (–16.73)
80 > S ≥ 60	0.35 (+0.1)	414 (–166)	71.38 (–28.62)
60 > S ≥ 40	0.4 (+0.15)	362 (–218)	62.41 (–37.56)
40 > S ≥ 0	0.45 (+0.2)	322 (–258)	55.52 (–44.48)

**Table 4:** $N_{x}$ depending on vehicle’s surrounding environment.

Speed (km/h)	HAR (${}^{{}^{\circ}}$)	$N_{x}$	$N_{x}$ Rate (%)
S ≥ 100	0.25 (–)	580 (–)	100 (–)
100 > S ≥ 80	0.3 (+0.05)	483 (–97)	83.23 (–16.73)
80 > S ≥ 60	0.35 (+0.1)	414 (–166)	71.38 (–28.62)
60 > S ≥ 40	0.4 (+0.15)	362 (–218)	62.41 (–37.56)
40 > S ≥ 0	0.45 (+0.2)	322 (–258)	55.52 (–44.48)
Sleep mode	0.50 (+0.25)	290 (–290)	50 (–50.00)

**Table 5:** Power consumption depending on $N_{x}$.

$N_{x}$	Supply Voltage (V)	AC (A)	APC (W)	Reduction Rate (%)
No-load	12.054	0.578	6.971	-
580	12.055	0.617	7.442	100 (–)
483	12.055	0.615	7.409	99.56 (–0.44)
414	12.055	0.609	7.347	98.72 (–1.28)
362	12.056	0.606	7.308	98.21 (–1.79)
322	12.056	0.605	7.289	97.95 (–2.05)
Sleep mode	12.057	0.593	7.154	96.14 (–3.86)

**Table 6:** Power consumption rate of laser diode (LD).

$N_{x}$	DAC (mA)	DAPC (mW)	PRR (%)
580	39.0	470.4	100 (–)
483	36.3	437.8	93.08 (–6.92)
414	31.1	375.4	79.80 (–20.20)
362	27.9	337.1	71.67 (–28.33)
322	26.3	317.9	67.57 (–32.43)
Sleep mode	15.1	183.0	38.91 (–61.09)

## Abbreviations

The following abbreviations are used in this manuscript:

LiDAR	light detection and ranging
HAR	horizontal angular resolution
LD	laser diode
NIR	near-infrared ray
ToF	time-of-flight
APD	avalanche photo diode
TDC	time-to-digital converter
MP	microprocessor
AMCW	amplitude-modulated continuous-wave
FMCW	frequency-modulated continuous-wave
MEMS	microelectromechanical systems
OPA	optical phased array
RADAR	radio detection and ranging
VAR	vertical angular resolution
BLDC	brushless direct current
VFoV	vertical field of view
PWM	pulse width modulation
TMR	internal timer
HFoV	horizontal field of view
SPI	serial peripheral interface
OSC	oscillator
BRR	BroadR-Reach ethernet
CAN	controller area network
ECU	engine control unit
PCL	point cloud library
AC	average current
APC	average power consumption
DAC	difference in average current
DAPC	difference in average power consumption
PRR	power reduction rate
